# Understanding “revolving door” patients in general practice: a qualitative study

**DOI:** 10.1186/1471-2296-15-33

**Published:** 2014-02-13

**Authors:** Andrea E Williamson, Kenneth Mullen, Philip Wilson

**Affiliations:** 1 General Practice and Primary Care, School of Medicine, College of MVLS, University of Glasgow, Glasgow, Scotland; 2 School of Medicine, College of MVLS, University of Glasgow, Glasgow, Scotland; 3 Centre for Rural Health, University of Aberdeen, Inverness, Scotland

## Abstract

**Background:**

‘Revolving door’ patients in general practice are repeatedly removed from general practitioners’ (GP) lists. This paper reports a qualitative portion of the first mixed methods study of these marginalised patients.

**Methods:**

We conducted qualitative semi-structured interviews with six practitioner services staff and six GPs in Scotland, utilizing Charmazian grounded theory to characterise ‘revolving door’ patients and their impact from professionals’ perspectives.

**Results:**

‘Revolving door’ patients were reported as having three necessary characteristics; they had unreasonable expectations, exhibited inappropriate behaviours and had unmet health needs. A range of boundary breaches were reported too when ‘revolving door’ patients interacted with NHS staff.

**Conclusions:**

We utilise the ‘sensitising concepts’ of legitimacy by drawing on literature about ‘good and bad’ patients and ‘dirty work designations.’ We relate these to the core work of general practice and explore the role that medical and moral schemas have in how health service professionals understand and work with ‘revolving door’ patients. We suggest this may have wider relevance for the problem doctor patient relationship literature.

## Background

We begin by exploring the current available evidence about ‘revolving door’ patients in general practice. We then present our findings from a grounded theory study of professional perspectives about these patients. Following on from this we utilise some theoretical ‘sensitising concepts’ to locate our results in the sociology literature which bring new insights into problematic doctor-patient relationships.

### Repeated removal from GP lists

General practices in the UK operate a list system which defines their patient population. ‘Revolving door’ patients in general practice are those who are repeatedly removed from general practitioners’ (GP) lists, for the reasons of break down in the doctor-patient relationship or violence. The definition excludes those removed because of geographical relocation. Being registered with a general practitioner (‘on a list’) is necessary to access most National Health Service (NHS) services. A stated aim of general practice is to provide coverage, continuity and quality of relationship for patients [1].

We reviewed studies of problem doctor-patient relationships in primary care to find out whether ‘revolving door’ patients had been investigated. Utilising a conceptual framework that maps the doctor patient relationship literature [2] there were four domains; the psychodynamic, the clinical- observational, social-psychological and the sociological. They included papers that have been influential in the field, especially Groves’ (1978) ‘hateful patient’ work [3], the literature about ‘heartsink’ patients in the UK [4,5], and the substantive literature about ‘frequent attenders’ in general practice [6]. One paper by a UK GP crossed the four domains and reviewed the ‘difficult patient’ literature from the 70’s and 80’s in general practice and other fields: Smith considered sociological, clinical observational and psychodynamic perspectives [7]. We also reviewed literature about ‘somatisers’ [8] and patients with ‘medically unexplained symptoms’ [9] because they were discussed in many of the papers included in these domains.

A number of investigators studied patient removals before the last iteration of the UK GP contract [10-21]. Evidence of patients who were repeatedly removed from GP lists was only explicitly considered in one paper that opted to exclude them from their analysis [10], one that described that some participants ‘found themselves being repeatedly removed and reallocated’ [19] and one that recommended management strategies for ‘revolving door’ patients without any consideration of their characteristics or the reasons for their repeated removal [16]. One additional study interviewed the GPs of patients who ‘revolved’ in and out of psychiatric hospital. They reported an overlap between these patients and ‘revolving door’ patients in general practice but did not explore this link further [22].

‘Revolving door’ patients thus seemed to be excluded both from the system of care and from the research literature, and merited further investigation.

This paper reports findings from a qualitative portion of a wider mixed methods study that was the first to specifically investigate repeated patient removals in general practice. Guided by the qualitative interviews with Practitioner Services staff and GPs with a specific role in working with ‘revolving door’ patients described shortly, we devised a definition of ‘revolving door’ patients as those removed four or more times from GP lists in seven years. From our analysis of routine NHS data on a sample of 555 ‘revolving door’ patients from 1999 to 2005 who were predominantly male (68%), had a mean age of 34 and a median deprivation decile of 9 (10 being the most deprived) we discovered these patients had high levels of psychiatric, addiction-related and physical morbidity. They also had much higher mortality rates when compared with the overall general practice population [23].

## Methods

### Participants

Following initial key informant contacts with Practitioner Services and Health Board managers about ‘revolving door’ patients, we sought and obtained ethics approval in 2006 (Oxford (B) NHS Research ethics committee, ID number 06/Q1605/74), and written consent from all participants. We purposively sampled and conducted six semi-structured interviews with Practitioner Services Division (PSD) staff who administered the GP registration system across Scotland. This was to capture the ideas and experiences of a registration administrator and a regional manager from each of the three regional offices in Scotland. The key informant PSD contacts had told us that despite having an administrative role in the NHS dealing with thousands of patient registrations per year, they knew ‘revolving door’ patients well mostly through telephone contacts with them. They were also enthusiastic about taking part in the study as they viewed this as an important problem that took up a lot of time and resource.

We also purposively sampled two GPs whose managerial or clinical role in the NHS meant they had in depth experience of working with ‘revolving door’ patients. One was both a GP and a manager of a large city health board primary care division and one had a clinical role in a service that worked specifically with ‘challenging’ patients.

Further semi-structured interviews were also conducted in 2010 with four GPs working in practices covering geographical areas where prevalence of ‘revolving door’ patients we found to be high. These four interviews followed our analysis of the Community Health Index (CHI) data on all patient removals in Scotland from 1999 to 2005 which showed a dramatic decline in the number of ‘revolving door’ patients being generated [23]. These GPs worked in two health board areas and across 3 towns and one city in the West of Scotland. The main role of three of the GPs was in practice, with each having GP training or a wider NHS management role. One GP participant was mainly a primary care manager.

Based on the literature on single episode patient removals we considered that to interview GPs with no specific role in working with ‘revolving door’ patients might lead to superficial accounts of professionally acceptable practice.

The GPs approached readily took part in the study. The interviews were conducted and audio-recorded by AEW. The topic guide covered the definition of a ‘revolving door’ patient, their characteristics, their impact, the reasons for their repeat removal, the importance of their existence, patients who had stopped revolving, future care for ‘revolving door’ patients, and for the 2010 interviews, why the numbers of patients had changed. Additional file 1 describes the detail of the topic guide in a table.

### Analysis

Our approach to the study was informed by the version of grounded theory attributable to Charmaz. Grounded theory was chosen because of its methodological advantages when exploring a new topic with limited theoretical underpinnings and the Charmazian version because it’s constructivist stance [24] was congruent with our epistemological perspective. The analysis of the transcribed interviews was conducted by AEW with the support of ATLAS Ti software. Data were coded using ‘incident coding,’ that is portions of data with meaning, and using ‘in-vivo’ codes, using the words of the participants. Many codes were generated by this process and the transcripts were read and re-read many times to ensure all codes were captured. Specific attention was paid to areas of text that were not coded and explicit consideration was given to why these portions were excluded including whether they may be data that contradicted the beliefs and views of the researcher. Higher order codes were then generated from grouping these initial codes by themes. Summaries of all the Practitioner Services (PSD) interview transcripts under these coding headings were reviewed by the PSD staff participants, and notes were made from follow up telephone discussions with them about these. No new themes were identified from this process. PW and KM reviewed the development of the codes following analysis of the GP participant interviews. Participant validation was not conducted with the GP interview transcripts. Some codes distinct from the PSD interviews were identified. Data saturation [24] was reached as judged by AEW, KM and PW at this stage and no further interviews were conducted.

The results were integrated in a dialectic way: that is they were compared to seek and explain differences between them [25]. They were considered to be analytically generalisable in that they helped to generate theories about ‘revolving door’ patients [26] and we utilised ‘sensitising concepts’ from the sociological theories described to support this [24].

## Results

### The three necessary characteristics of ‘revolving door’ patients

The research participants were unanimous when they described three characteristics that all patients shared in order that that they became ‘revolving door’ patients.

#### Unreasonable expectations

The first was that participants perceived that all ‘revolving door’ patients had unreasonable expectations of the Health Service. This could be expressed in a range of ways. An example was that patients requested consultations for perceived health needs very frequently, as illustrated by this quote:

‘literally couldn’t pass the health centre on the bus without stopping and coming in [expecting to be seen] and that was very difficult really but…[the] family…was just riddled with problems’ GP6, (General Practitioner participant 6).

Some participants viewed this as linked to a lack of ability to distinguish between minor and major illnesses.

Participants perceived that the subsequent response ‘revolving door’ patients expected from the practice could be unreasonable too; including having unrealistic preferences for one GP ,making repeated requests for 999 ambulances, and repeated house call requests, as described in this quote:

‘He [the visiting GP] played the piano in the house and he wasn’t asked to, switched the light on and she didn’t give permission to do that in her bedroom. But she will find fault with anybody because she wants someone particular; there is only one [GP] that she likes particularly…so everybody that goes in there she finds fault with…she calls out ambulances all the time, day and night, she makes 999 calls, which the practice inevitably get involved in, and she is exceedingly demanding. And she demands house calls all the time; and then she won't open the door because she is having her tea’PS3 (practitioner services staff participant 3).

Some patients phoned the practice or other services repeatedly after they had just been seen. Under-pinning these experiences were the practice’s view that they could not hope to meet the patient’s perceived needs:

‘You start off, and you try and sort out some of their problems; but then you realise with some of them; unless there's a change in their perceptions and so on, things aren’t going to get better. Some of them have got chronic diseases they've just simply not accepted. How do you get them to realise you aren’t going to get better; you’re always going to have some sort of disabilities? Are you going to have changing perceptions of what we expect here, what we can and cannot try for you there?’ GP5.

#### Inappropriate boundaries of behaviour

The second necessary characteristic that ‘revolving door’ patients had was the perception that their boundaries of behaviour were difficult for others to accept. This was described as becoming apparent as soon as the patient began to interact with the practice and they made health staff feel threatened or exasperated, including receptionists and Practitioner Services staff who administered the registration system. Exasperation was bound up with perceived unreasonable demand when the patient seemed unwilling or unable to change their pattern of behaviour relating to this.

Participants believed that patients who were persistently abusive or impolite to reception staff and health professionals, became ‘revolving door’ patients. Some ‘revolving door’ patients struggled to control their anger and blew up easily as described in this quote:

‘Yes the ones [‘revolving door’ patients] that I have met have a tendency to become very quickly verbally abusive, and I think that’s why people want to back away from them. So I think that would be their main common characteristic. On the phone as well they quickly become out of order with their language and insults, inappropriate insults very rapidly.’ GP1.

#### Unmet health needs

The third necessary characteristic that the participants reported was that ‘revolving door’ patients themselves felt that they had health needs that required to be met. These may be physical, psychological, or needs related to the medical aspects of benefits or insurance. This was important because otherwise patients would not continue contact with general practice and might simply avoid registering with a new practice once removed. Participants described the health problems that they perceived ‘revolving door’ patients to have. Practitioner Services participants knew about many ‘revolving door’ patients who they viewed as having high dependency needs such as being housebound and requiring regular nursing input, or having agoraphobia and requiring house calls.

Participants described ‘revolving door’ patients whom they believed to have mental health problems. This was articulated in different ways by Practitioner Services and GP participants; the first describing how patients interacted but not explaining behaviour in mental illness terms, the second providing mental health diagnoses. Practitioner Services participants described patients who behaved bizarrely, seemed to have conversations with themselves, were demanding, appeared delusional, and even displayed inappropriate sexual behaviour. Here is an example of a description:

‘This patient is a bit; I use words like delusional, and I am not medically qualified, but she has odd ideas about patient data and doesn’t want to go to the practice across the road because of some programme she heard on radio four which suggested that obviously they would tell everybody all this information sharing within the NHS. And she feels that there is no privacy. She doesn’t want to go to the practice that’s nearest to her because she doesn’t want her neighbours and everything knowing all her business which of course the GP practice is going to tell them. She refers to things she has read and to consultants that she knows personally who have given her advice about this, that and the other, and she is quite difficult. And she makes accusations against the practice when she is there which is quite difficult for them and unsubstantiated. And therefore ends up going to the next one. They get fed up with her as well, she goes back.’ PS3.

The GP participants considered that the majority of ‘revolving door’ patients had personality disorders, were likely to have been discharged from psychiatry services, and that general practice was ill equipped to work effectively with them:

‘…the last one we had it was particularly frequent, inappropriate house calls; demanding; aggressive; playing one person off against the other; being abusive verbally to staff. That was the last one we had; it was somebody who had learning disabilities and was in a home, and refused to cooperate with all treatments. She used to have numerous complaints and was over investigated…. And her case notes were horrendous; large part of them were personality problems.’ GP5.

Patients who were anxious and expressed their symptoms through physical complaints and health seeking behaviour were viewed as a subset of these patients. GPs also gave examples of a few patients who were described as having milder learning impairments and some described patients with major mental illnesses who became ‘revolving door’ patients as described in this example:

‘One patient was moved on a few times when she had several periods of actual physical aggression when she was psychotic. She was schizophrenic and she had quite a few serious assaults actually.’ GP1.

### Deviant case analysis

There were three areas of data that stood out when thinking about the necessary characteristics of ‘revolving door’ patients which relate to participants perceptions about ‘revolving door’ patients health problems.

#### Alcohol dependent patients

The first was that patients with alcohol dependency problems were typically thought by the participants not to become ‘revolving door’ patients. GP participants felt that this may be because GPs were able to form reasonable doctor-patient relationships with most alcohol dependent patients. The perception was that they tended to have periods of relative stability and positive contacts with general practice in between more chaotic times and even in those chaotic times made more reasonable demands of general practice:

‘R:…if someone is merely drinking themselves to death at home, they don’t want help, there's not a lot I can do. Someone who is bouncing out of hospital up and down to casualty, fine; there's nothing I can do about it, we can offer you X, Y, Z but if you don’t want it you don’t want it. The ones who will cause problems, who annoy you are the ones who, you know are repeatedly phoning you out late at night and so on, most of them aren't great; there's addictions services they can see. What I reckon is that tolerance has gone up and up and up; what will we do with their physical problems? Most of them come in and are pleasant enough to you, they will tell you what life is, and what they want; their benzos [benzodiazepines] and all that; and this is going to make it so much better; and they are going to cure themselves and so on.

I: So their interaction’s ok?

R: By and large- unless there's underlying problems there -and most of them; if you; where we work; if you can’t always deal with the alcohol problems you would put a lot of people off the list!’ GP5.

This however contradicts the evidence from the routine health service data about the sample of ‘revolving door’ patients reported in our previous paper: alcohol dependency was an important condition for many patients [23]. It may be that the sample of patients we studied did not raise their alcohol problems with their successive GPs so it was a hidden problem. This is possible; however alcohol dependency was actively raised and discarded by participants as a reason for patients’ ‘revolving’.

#### Major mental health problems

The GP participants were clear when asked to describe the characteristics of ‘revolving door’ patients that patients with mental health problems that were ‘severe and enduring’ did not ‘revolve’ even though they may be challenging to care for and interact with the practice in similar ways. However one example of a patient who was a ‘revolving door’ patient and had a schizophrenia diagnosis was described. Like the patients with problem alcohol use, or even with a learning disability diagnosis, it may be that the GP participants focussed on expectations and behaviours they did not attribute to problem alcohol use, learning disability or schizophrenia when they decide to repeatedly remove patients.

#### Problem drug using patients

The third unmet health need was the area of problem drug use. Historically patients with problem drug use were reported by participants as the majority of ‘revolving door’ patients before addiction treatments and services developed. This pattern changed at different times in different Health Board areas as treatment services were set up and became available. GPs gained knowledge and skills about treating problem drug use and they began to prescribe maintenance methadone treatment. For the GP participants, these were the main reasons for the reduction over time in numbers of patients with drug problems becoming and remaining ‘revolving door’. This change in the approach GPs had in working with patients meant that drug using patients’ behaviour changed. Practitioner Services participants generally agreed with this, but some took the view that many of the patients with problem drug use were getting older, becoming physically more unwell and maybe quite naturally their perceived aggression and drug seeking behaviour had settled down.

‘But I think my worst [‘revolving door’ patient] had serious problems internally and he had to go in to hospital. And when he was discharged I think- now- the guy is pretty ill and he had been a drug addict since he was about fifteen. And I think he’s something like fifty now; and only up to about three years ago I finally got rid of him [the patient stopped ‘revolving’]…But like that; a lot of them are getting older now and I think they are dying off; or if they just can't take it the same; so I don’t know if that’s part of it as well.’ PS4.

There was a perception by some participants that some GPs were being ‘inappropriate’ now, in not meeting the treatment needs of drug misusing patients.

### Professional roles and boundaries

Both practitioner services and GP respondents felt there were neither the structures in place nor did they have the professional expertise to work effectively with ‘revolving door’ patients. Bound up with the perceived unreasonable expectations and inappropriate behaviours that participants felt all ‘revolving door’ patients had was the feeling they crossed many of the normative boundaries that most patients obeyed.

They were perceived as taking up too much time- participants described ‘revolving door’ patients as being high workload patients. For the Practitioner Services participants they described the frequent administrative process of registration, removal and reinstatement. A large amount of written correspondence was generated due to complaints and hospital letters addressed to the patients’ previous GPs that were re-routed to Practitioner Services and had to be filed. There were frequent phone calls from patients and practices. For the GPs, ‘revolving door’ patients often took up time because of the need to respond to their demands, behaviour or unmet health needs. This GP respondent describes the consultations with one ‘revolving door’ patient:

‘And he frequently gets fixed ideas about things and persistently asks for them…so he keeps going on about that, so its quite difficult to keep the consultation to a reasonable length of time…I can see that it would be very frustrating to deal with because you would be running later and later and wanting him to go; he frequently comes back in as well. You know, you think he has gone and then he will come back and asks more…’ GP1.

Both practitioner services and GP participants described boundary strategies they used to attempt to contain ‘revolving door’ patients’ demands and behaviour, such as regular pre-planned appointments or identifying one professional who worked with the patient. Despite these they described many examples where both they and others felt the systems in the health service were not in place to work effectively with these patients.

Moreover Practitioner Services participants described ‘revolving door’ patients phoning seeking medical advice that they were not able to give. They often worried that the general advice they did give about accessing appropriate health services might be wrong or might make the patient’s problem worse.

‘I'm not a care worker and I don’t know what to say to you. I mean I can listen, yeah, and I can sympathize, and I can make suggestions, but it’s just a case of who to phone rather than anything else. I mean, I'm not medical; so you wouldn’t want to say anything that would affect her as well. So you're trying to be neutral and even that itself can be quite difficult; trying to get off the phone without sounding as though you just can’t be bothered with her.’ PS2.

GP participants too described their feeling of lack of professional expertise:

‘So the psychiatrists, the psychologists, community psychiatric nurses really don’t want to know and that feeling of abandonment is quite difficult to deal with. And so you think, ‘oh no, what am I going to do with this person?’ They clearly need help and I don’t actually feel that qualified to be able to deal with it but nobody else is willing to engage with them either. And sometimes there are people who are willing to try but then the patient won't engage with them either. You think ‘oh please do something that I am advising’ but they won't.’ GP1.

The participants talked about having to deal with a lot of negative emotions generated in response to ‘revolving door’ patients. Practitioner Services participants got ‘frustrated’, ‘fed up’, ‘annoyed’, and sometimes ‘angry’ with ‘revolving door’ patients. This was partly because they found them difficult to deal with but also because they felt their boundary breaches prevented them getting on with other work. Also over time some of the Practitioner Services participants came to the conclusion that part of the toll was that no matter what amount of time they spent on the phone, the issues and perspectives of the patients were unchanging.

Practitioner Services staff also described practices having significant memories of ‘revolving door’ patients and their experiences with them; to the point where they expressed anger when ‘revolving door’ patients were assigned to the practice again.

One GP participant felt that information given by practices to Practitioner Services about ‘revolving door’ patients offered a form of exorcism or catharsis, a boundary breach on their part:

‘Occasionally the GPs would write to Practitioner Services explaining all their reasons; and that’s a bit of a breach of confidentiality actually; because this was an administrative function. But that was the way of it. They were so exercised by this patient that they wrote in and gave them chapter and verse as to how they were putting them off; but there wasn’t a kind of safe haven or some area for discussion about how these patients were handled.’ GP2.

For some ‘revolving door’ patients there was evidence reported that patients displayed similar behaviours with other agencies they interacted with; the local authority, housing services, social work, for example, as illustrated in the following quote:

‘I have a gentleman on the go at the moment [being repeatedly removed] who has made accusations of various people being racist and there was going to be a sort of meeting arranged with social work and the race equalities board and things like that; and there were a lot of people involved; a lot of third parties involved in dealing with this gentleman we were speaking to. So it was quite obvious he had issues with a lot of people; about things, so sometimes there might be social work involvement or another third party….’ PS3.

## Discussion

Having presented our results we now explore the ‘sensitising concepts’ that we used to inform our further theory development and conclusions. We utilise theories about the role of legitimacy [27], ‘good and bad’ patients [28], and ‘dirty work designations’ [29,30] following Shaw’s study of ‘revolving door’ psychiatric patients. Shaw discussed ‘moral judgements’, ‘responsibility’ and other factors that might impact on legitimacy [22]. We extend this, building on the work of Strong [30] by developing the idea that medical and moral schemas have a vital role in how GPs function in their core work.

### The role of legitimacy

Kelly and May (1982) undertook a review of ‘good and bad’ patients in the nursing literature and in key sociology texts. Their critique highlighted some important points to consider when generating theories from the participant interviews in this study. They began by describing the illnesses, symptoms, behaviours, perceived patient attitudes and judgements of staff; strikingly similar to the themes and categories identified in this paper. They went on to describe the discrepancies and contradictions between the ‘good and bad’ patient studies and conclude that the topic lacked external validity. They surmised this could in part be explained by the range of research tools used; but most importantly because the concepts used were not rigorously defined. The studies explored staff opinions about patients, and made assumptions about the meanings, for example, of ‘aggressive’ or ‘inappropriate’. The studies seemed unquestioningly to locate ‘good and bad’ characteristics in the patients; rather than in the professionals’ opinions about the patients; these opinions are treated as objective facts. Kelly and May concluded that causality and consequence were also assumed, considered in a linear simplistic fashion and the links between these not made explicit [28]. This is an important reminder for the context of this study; the labels applied should not be viewed in the same structuralist manner in which for example a clinical diagnosis might be applied.

In line with Kelly and May’s review of the ‘good and bad’ patient literature NHS staff believed ‘revolving door’ patients’ difficulties were located within the patient: they had unreasonable expectations and inappropriate behaviour. They viewed these as being beyond the expected norms of patient behaviour within the doctor patient relationship. This echoes Stokes’ qualitative work about patient removals which concluded these happened because patients broke the unwritten rules of the doctor-patient relationship [18]. A future paper using psychological theories will re-examine the role unwritten rules might have for ‘revolving door’ patients.

Another important aspect of Kelly and May’s review was that they thought carefully about the value based assumption they considered to permeate the literature; that ‘good and bad’ patients are a problem to be fixed and that the explanation for their poor care is the fault of poor professionalism. This played a role in the initial conception of this research study and in our motivation for choosing the topic. Kelly and May argued that the literature failed to consider that professionals may have understandable reasons for so labelling patients; because such patients actually do make their work difficult. They postulated too that, with few exceptions, an intensely individualistic view of the issue was dominant; the social setting was not considered and a rigid structuralist approach to theorising was applied across the literature. Kelly and May sought to revise this and used an interactionist approach building on the background of Parson's work on the sick role. Their central conclusion was to propose that in the ‘good and bad’ patient literature:

‘it is in the process of providing or withholding legitimation that patients come to be defined as good and bad’ [28].

They expanded on this conclusion in a follow up paper; patients are good patients if they uphold the role of the health professional; they are bad if they negate it [27].

The concept of ‘dirty work designations’ extends the concept of legitimacy. First described by Everett Hughes in a series of studies from the 1950's and 1960s it has been used to examine work roles in a number of occupations and settings including in health [23]. Emerson and Pollner in their study of a community mental health team in the USA; described ‘dirty work designations’ as seeming to have significance at several levels:

‘On one level the designation of a task as dirty work may be understood as a more or less faithful portrayal of its odious and onerous qualities…on an analytical level dirty work designations implicate the perspective of the worker as much as they do the quality of the work…one occupation's dirty work can be another's sought and fought for prerogative… while dirty work designations are the product of a particular perspective they are the means through which the perspective is enacted and perpetuated…dirty work reaffirms the legitimacy of the occupational moral order that has been blemished’ [29].

This emphasised that dirty work, like the interactionist interpretation of ‘good and bad’ patients, embodies a mismatch between what the doctor sees as his/her legitimate work and the problem the patient presents [29].

In his study of ‘dirty work’, GPs and alcohol dependent patients, Strong added a further dimension to legitimacy by viewing ‘dirty work’ as a function of the patient's ability to negate the professional's self-perceived core roles:

‘This fundamental disjunction with the role-relationship seems a more plausible account of why alcoholics should be dirty work than that of traditional morality or faulty education.’ [30]

#### The core work of general practice

As the context for this study was general practice, we will consider the core role of general practice, and thus the boundaries of its legitimate work. There is consensus from the literature that core work is in two areas. The first is the technical biomedical aspect of care that GPs and practices deliver. This includes the range of problems relating to health and health care that GPs view they have a role in solving or signposting to others to do so. The second is the centrality of the relationship GPs have with their patients, the practitioner patient relationship being a focus of care [1,31-33].

### Medical and moral schemas

Understanding how the technical biomedical aspect of care might relate to theory generation about ‘revolving door’ patients in general practice is helped further by Strong’s study. He drew on the work of Chalfant and Kurtz who studied social workers and their alcohol dependent clients in the 1960s. He postulated that, as for social workers, doctors’ schemas about how they conceptualise their work are important when deciding what is and is not ‘dirty work’.

‘we are currently in the middle of a long term shift from a moral to a medical theory of alcoholism and that social workers- and possibly other professionals too- apply elements from both schemas. Thus, although they are morally hostile in some ways to alcoholics, they are not entirely so and in the long run these irrational elements will fade’ [30].

We reviewed Strong’s use of schemas. In cognitive psychology schemas are ‘organised packets of information about the world, events or people that are stored in long term memory’ that have been studied in relation to narrative memory and recall. Schema theory has been criticised in that field, because the knowledge contained in schemas is difficult to describe, and it is not clear when a particular schema might be activated [34]. In clinical psychology, schemas are a ‘central component’ of cognitive theories of personality development, and are defined as a ‘consistent internal structure, used as a template to organise new information.’ They are viewed as the “generals’ of the information processing system and govern all other systems’ [35]. Schema theory has also been used to develop a conceptual framework for a social theory of intercultural communication and we found this framework for schema theory helped us explore Strong’s concept of medical and moral schemas. ‘Cultural schemas’ relate to packets of information about individual’s experiences within social groups or cultures. They are

‘generalised collections of the knowledge that we store in memory through experiences in our own culture. Cultural schemas contain general information about familiar situations and behavioural rules as well as information about ourselves and people around us. Cultural schemas also contain knowledge about facts we have been taught in school or strategies for problem solving and emotional or affective experiences that are often found in our culture. These cultural schemas are linked together into related systems constructing a complex cognitive structure that underlies our behaviour’ [36].

We interpreted Strong’s concept of a medical schema to incorporate the knowledge and experiences that health professionals use to inform their understanding of attitudes, behaviours and illnesses. This is influenced by what is learned in medical training, in professional development over a career including clinical practice context, the influence of colleagues’ practice and patients encountered. This changes over time too as medical philosophy and medical knowledge changes. There are also medical schemas of understanding influenced by medical knowledge that have everyday significance in general society and that lay people hold. We interpreted the concept of moral schemas to mean the understanding of attitudes, behaviours and illnesses based on the dominant philosophies and social values of society that also change over time. Doctors are also members of society so are influenced by moral schemas as well as the medical ones they hold, so reciprocal for both lay and medical members of society. These medical and moral schemas of understanding about attitudes, behaviours, and illnesses are locked into the ways that GP’s, other professionals in general practice and practitioner services staff understand the technical biomedical sphere-the health problems or behaviours that patients bring to them. Crucially this further shapes their expectations of the interactions they have with patients within the doctor patient relationship or the GP practice. If professionals can locate the explanation for an attitude, behaviour or health presentation within a medical schema of understanding, they will tolerate patients who do not obey the unwritten rules of the doctor patient relationship. If however these are understood within a moral schema then they are more likely to be understood as being about the patient himself or herself, as a problem located within the patient, not about their illness.

‘someone with major psychotic illness; mental health have got a lot of support services for that, intervention stuff but behaviour acceptable, paradoxically they may have little insight but you see that’s their, you can identify this person as mentally ill; and so you treat it accordingly. Someone with personality disorder with very complex diagnoses that often take ages; you are thinking ‘you are just at it; you are just out to deliberately frustrate our efforts' as it were. And I think, someone who has got a psychotic illness will be frustrating their efforts perhaps but done through their illness. There's a perception of personality disorder, frustrating all your efforts and so on, they possibly out of badness sometimes crosses- and you will get frustrated with them.’ GP5.

There was evidence from the GP participants’ accounts that they consider psychiatric illness in general practice with a medical schema of understanding that is distinct from what might reflect a lay medical schema described by the Practitioner Services participants. The GP participants described one group as patients who have serious mental health problems, represented (using the commonly used clinical phrase) as having ‘severe and enduring mental illness’ and the other group of patients as having a ‘personality disorder’. However although all the GPs described patients with personality disorder as being mentally ill but there was evidence of a moral schema of understanding too, expressed in the quote above about ideas about how much responsibility patients were able to take for their own actions. This mirrored the way in which patients with a personality disorder diagnosis were reported as often treated by psychiatry services.

It is apparent from these data that there is a medical schema of understanding for patients with a diagnosis of severe and enduring psychiatric illness such as psychosis. This is likely to be due to the prevailing diagnostic frameworks and the availability of widely accepted treatments. The conceptual framework for understanding personality disorder however remains contested and the evidence base for effective treatments has still not been absorbed into mainstream psychiatric practice [37]. The medical professionals in this study also reflected this by applying the medical diagnosis of ‘personality disorder’ but using a moral schema to conceptualise the behaviour of patients to whom they apply this diagnosis.

Alcohol dependency and drug misuse demonstrate the central importance that medical and moral schemas of understanding have when considering these theories about ‘revolving door’ patients. The alcohol accounts support Strong’s hypothesis that alcohol dependency has undergone a shift from a moral to a medical schema of understanding in society. The professionals in this study considered and discarded alcohol dependence as an explanation for patients being ‘revolving door’ patients. So [22] being alcohol dependent is no longer seen by doctors as a moral problem-’dirty work’ so a problem that means a patient can be struck off their list- but as a health problem within their biomedical technical role. So being alcohol dependent is not used as the explanation for why patients becoming “revolving door”, although the quantitative data tells us a proportion were. They use other aspects of patients’ presenting problems to label them ‘difficult’ within a moral schema, and for them to be categorised as ‘revolving door’ patients. Patients can have multiple identified health problems, but it is the ones that are identified as being within a moral schema and a dominant issue that leads patients to ‘revolve’.

It is evident from this study that problem drug use has undergone a similar shift in status as the professional participants described the transformation in drug use treatments and services:

‘..it really kicked off about 92, 93, a lot of people started appearing, we had no training in it, we didn’t know what to do. GPs didn’t know what to do, there was no hospital base, there was an alcohol service but there wasn’t a drug service and more people were appearing and we didn’t know what to do with them. Over time, some of these patients became so insistent and abusive and demanding of practices that eventually they would, we would try our best with them but they would cross a line. They would go to another practice, they would repeat the same behaviour, they would cross a line and eventually, they’ve gone round all the practices in the area and their behaviour would still continue.…when we got a drugs service which was effective and people were getting into treatment, and they were being stabilised, then a lot of these patients problems disappeared’ GP4.

Many participants did locate drug misusing ‘revolving door’ patients’ difficulties as being about the service not previously responding to their needs and having changed, so now within a medical schema, not located as an inherent characteristic of the patient. There was however evidence of a moral schemas of understanding too from some participants:

‘…more people are deciding that perhaps it is manageable within primary care so that was the first step; methadone. We started finding methadone; because there was a lot of people dying. I thought well I know they are obnoxious and a pain; but they are someone’s mother someone’s daughter. And there's no doubt that methadone is sedating, there's no 2 ways about it, it does sedate you, you can argue whether it’s a good thing or a bad thing; it makes life infinitely, infinitely, more manageable’ GP5.

One possible explanation for this difference between participants is that some may have a medical schema for understanding problem drug use but some may have elements of a moral schema. Could problem drug use be going through a similar transition to that reported by Strong over 30 years ago with alcohol dependency? Similarly what status does personality disorder have in relation to transition?

All the participants were clear that ‘revolving door’ patients made their professional life difficult in the range of ways described above. This resonates with Kelly and May’s review (1982) of the ‘good and bad’ patient literature and helps to place the topic in its social context. However our stance is that ‘revolving door’ patients’ attitudes, behaviours and health presentations can be framed within a GP’s medical schema of understanding. So it is our view that these negative attributes are operating within the doctor-patient relationship, rather than as inherent to ‘revolving door’ patients themselves. We have presented evidence of how a change in professional perceptions of alcohol and problem drug use changed patients’ behaviour.

We were struck by the relevance our theoretical perspectives may have for the wider problem doctor patient literature that we reviewed. Each provided evidence of patients threatening the legitimacy of the core work of general practice by having elements of a moral schema of understanding for interpreting their behaviour or health presentations. For example ‘heartsink’ patients, some groups of patients who were ‘frequent attenders’, patients with ‘medically unexplained symptoms’ threatened the legitimacy of the doctor’s technical bio-medical care by presenting with problems that GPs could not fit within a medical schema so their care was ‘dirty work’. The patients categorised in the psychodynamic literature we reviewed, for examples in Groves (1978) paper on the ‘hateful patient’, and features of the presentations of patients in the other literature areas were understood within a moral schema because moral censure was applied to their behaviour. This perspective provides a unifying theory with which to understand a diverse body of literature and may provide future helpful insights into conceptualising the issue of problem doctor-patient relationships.

## Conclusions

In our study of ‘revolving door’ patients in general practice we have described the necessary characteristics of ‘revolving door’ patients. Participants were unanimous in that patients had unreasonable expectations, exhibited inappropriate behaviours and perceived themselves as having unmet health needs. Professional roles and boundaries were important and a range of boundary breaches were reported when ‘revolving door’ patients interacted with NHS staff, both in general practice and in the administrative setting of Practitioner Services who managed GP registrations. They took up time, led staff to believe they did not have sufficient expertise to deal with them and took a significant emotional toll. Staff described boundary strategies they employed and also times when staff found themselves breaching boundaries. We discussed our findings using the ‘sensitising concept’ of legitimacy by drawing on the literature about ‘good and bad’ patients and ‘dirty work designations’. We related this to the core work of general practice which is about providing technical biomedical care to patients and a positive doctor-patient relationship.

Following Strong (1980) we found that medical and moral schemas about attitudes, behaviours and health presentations were key to understanding why ‘revolving door’ patients challenge the legitimacy of GPs core work. We have shown that from professional perceptions about these, ‘revolving door’ patients challenge the technical biomedical role of GPs. We used the examples of psychosis, personality disorder, alcohol dependency and drug misuse to illustrate this and suggested that the role of legitimacy, utilising medical and moral schemas, might provide a unifying theory for the problem doctor-patient relationship literature.

In our analysis we located the social context for our findings and theory development in the doctor-patient relationship rather than as negative characteristics inherent to ‘revolving door’ patients. However we are now more aligned to the conclusion put forward by Kelly and May (1982) in their review of ‘good and bad’ patients: that the generation of ‘revolving door’ patients in general practice is not simply the fault of poor professionalism. Our analysis lends insight into why GPs took the extreme action of repeatedly removing patients from their practice lists. We also hope that this paper will prompt clinicians and academics to consider how the schemas they use, to understand the attitudes, behaviours and health presentations of patients they treat or conduct research with, impact on their everyday work practice.

## Competing interests

The authors declare that they have no competing interests.

## Authors’ contributions

AEW conceived the study design with supervision from PW and KM and conducted the interviews. Analysis was carried out by AEW and KM and PW reviewed the coding development. AEW drafted the manuscript with input from KM and PW. All authors read and approved the final manuscript.

## Pre-publication history

The pre-publication history for this paper can be accessed here:

http://www.biomedcentral.com/1471-2296/15/33/prepub

## Supplementary Material

Additional file 1: Table S1 Topic guide summary for the professional participant interviews. Click here for file
