# Supplementary material for: Understanding “revolving door” patients in general practice: a qualitative study
Source: BMC Fam Pract. 2014 Feb 13;15:33. doi: 10.1186/1471-2296-15-33 (PMC3930014; doi:10.1186/1471-2296-15-33)
Supplement: Additional file 1: Table S1 — Topic guide summary for the professional participant interviews. [file 1471-2296-15-33-S1.docx]

**Appendix 1**

Table 1 topic guide summary for the professional participant interviews

| Definition of a “revolving door” patients | Probe meaning  Circumstances and influences on their production |
| --- | --- |
| Characteristics of “revolving door” patients | Describe last case, typical case, other types, those that stand out, why?  Common characteristics described, circumstances, behaviours, attitudes  Those who don’t fit the above? |
| Impact on “revolving door” patients | Access to health care, quality of health care, their health, on other patients? |
| Reasons for repeat removal | Why remove? Who removes? Describe these practices, what precipitates removal? Why keep patients on? |
| Importance of the existence of “revolving door” patients | For participants, for patients, for the health service |
| Patients who have stopped revolving | What changed? |
| Apparent disappearance of “revolving door” patients^[[1]](#footnote-1)^ | What happened? Key influences on practice, locality, health board, nationally |
| Future | Suggestions for change |

1. Included for the 4 GP participant interviews conducted in 2010 [↑](#footnote-ref-1)
